# Leadership for AI Transformation in Health Care Organization: Scoping Review

**DOI:** 10.2196/54556

**Published:** 2024-08-14

**Authors:** Abi Sriharan, Nigar Sekercioglu, Cheryl Mitchell, Senthujan Senkaiahliyan, Attila Hertelendy, Tracy Porter, Jane Banaszak-Holl

**Affiliations:** 1 Krembil Centre for Health Management and Leadership Schulich School of Business York University Toronto, ON Canada; 2 Institute for Health Policy, Management and Evaluation Dalla Lana School of Public Health University of Toronto Toronto, ON Canada; 3 Gustavson School of Business University of Victoria Victoria, ON Canada; 4 College of Business Florida International University Florida, FL United States; 5 Department of Management Cleveland State University Cleveland, OH United States; 6 Department of Health Services Administration School of Health Professions University of Alabama Birmingham Birmingham, OH United States

**Keywords:** AI implementation, innovation, health care, leadership, AI, artificial intelligence, management, organization, health care organization, strategy

## Abstract

**Background:**

The leaders of health care organizations are grappling with rising expenses and surging demands for health services. In response, they are increasingly embracing artificial intelligence (AI) technologies to improve patient care delivery, alleviate operational burdens, and efficiently improve health care safety and quality.

**Objective:**

In this paper, we map the current literature and synthesize insights on the role of leadership in driving AI transformation within health care organizations.

**Methods:**

We conducted a comprehensive search across several databases, including MEDLINE (via Ovid), PsycINFO (via Ovid), CINAHL (via EBSCO), Business Source Premier (via EBSCO), and Canadian Business & Current Affairs (via ProQuest), spanning articles published from 2015 to June 2023 discussing AI transformation within the health care sector. Specifically, we focused on empirical studies with a particular emphasis on leadership. We used an inductive, thematic analysis approach to qualitatively map the evidence. The findings were reported in accordance with the PRISMA-ScR (Preferred Reporting Items for Systematic Reviews and Meta-Analysis extension for Scoping Reviews) guidelines.

**Results:**

A comprehensive review of 2813 unique abstracts led to the retrieval of 97 full-text articles, with 22 included for detailed assessment. Our literature mapping reveals that successful AI integration within healthcare organizations requires leadership engagement across technological, strategic, operational, and organizational domains. Leaders must demonstrate a blend of technical expertise, adaptive strategies, and strong interpersonal skills to navigate the dynamic healthcare landscape shaped by complex regulatory, technological, and organizational factors.

**Conclusions:**

In conclusion, leading AI transformation in healthcare requires a multidimensional approach, with leadership across technological, strategic, operational, and organizational domains. Organizations should implement a comprehensive leadership development strategy, including targeted training and cross-functional collaboration, to equip leaders with the skills needed for AI integration. Additionally, when upskilling or recruiting AI talent, priority should be given to individuals with a strong mix of technical expertise, adaptive capacity, and interpersonal acumen, enabling them to navigate the unique complexities of the healthcare environment.

## Introduction

### Artificial Intelligence in Health Care: Overview

Artificial intelligence (AI) technologies have gained significant momentum in health care, presenting a transformative potential across clinical processes, operational efficiency, decision-making, and workforce optimization [1-3]. The global AI market is projected to shift from US $14.6 billion in 2023 to a formidable estimate of US $102.7 billion by 2028 [4], unveiling a dynamic transformation of unprecedented scale. This investment, coupled with the engagement of nontraditional health care players such as Microsoft, Google, and Amazon and the convergence of technological prowess and health care innovation signaled by generative AI, will place the trajectory of AI in health care in a state of exponential growth [5].

Current investments in health care AI predominantly center on bolstering data capacity, enhancing computational power, and advancing methodological innovations in AI. This includes developing and testing AI models and algorithms tailored for precision medicine, drug discovery, clinical decision-making support, public health surveillance, operational optimization, and process improvement [6,7]. Notably, between August 2022 and July 2023, there were over 150 submissions of drug and biological applications incorporating AI and machine learning components to the US Food and Drug Administration, encompassing a wide array of therapeutic domains and developmental stages [8].

Yet the seamless integration of AI technologies into health care organizational settings presents a multifaceted challenge for health care leaders. This challenge arises from several factors, including the complex nature of AI models, the rapid pace of technological advancement, the imperative of regulatory adherence, ethical concerns surrounding data security and privacy, the risk of perpetuating racial and ethnic biases in data, the necessity of prioritizing human-centric approaches to patient care, and the intricate clinical workflows that must be navigated [9-15]. Furthermore, health care leaders are facing critical and intricate strategic decisions. They must discern which AI solutions merit investment while weighing the merits of in-house development against strategic partnerships with external vendors. Selecting the right vendors and defining the scope of collaboration is pivotal, as is devising a sustainable funding strategy to support both initial development and continuous innovation. Furthermore, they must confront the crucial question of whether to bring in new AI talent or bolster the expertise of their current workforce through upskilling. Each of these decisions will shape the trajectory of health care organizations as they navigate this transformative era. A report by Bain in 2023 revealed that although 75% of surveyed health system executives recognize AI’s potential to reshape the health care industry, only 6% have established concrete strategies related to AI [16].

The lack of strategy and strategic failures in AI integration not only have financial consequences for organizations but also erode trust among patients, providers, and organizations [17]. A prominent example is the collaboration between MD Anderson and IBM Watson, aimed at leveraging IBM Watson’s cognitive capabilities to combat cancer. This ambitious endeavor, however, incurred a substantial financial toll of over US $62 million for MD Anderson because of setbacks in clinical implementation [18].

Despite a growing body of AI literature, including toolkits such as Canada Health Infoway’s “Toolkit for AI Implementers” [19] and guidance from the US Department of Health and Human Services’ AI Task Force [20] and the UK National Strategy for AI in Health and Social Care [21], there is still insufficient scholarly attention on how leadership behavior guides AI transformation in health care. Existing reviews focus on AI in medical education [22,23], workforce impact [24], applications in clinical medicine [13,25], barriers to implementation [26,27], and ethical considerations [28,29]. However, no systematic mapping of empirical literature has clarified our understanding of leadership or identified gaps in research. Understanding leadership behavior is crucial for health care organizations considering AI because effective leadership shapes the strategic direction, adoption, and successful implementation of AI technologies.

### Research Aim

To address this research gap and to establish a future research agenda this scoping review study aims to address two primary questions: (1) What role does leadership play in AI transformation within health care? and (2) What approaches can health care organizations use to empower their leaders in facilitating AI transformation?

## Methods

### Research Approach

This review follows scoping review methodology [30] to identify and analyze the current literature and report results following the PRISMA-ScR (Preferred Reporting Items for Systematic Reviews and Meta-Analyses for Scoping Reviews; Multimedia Appendix 1) guidelines [31].

### Key Definitions

In the context of this study, AI refers to combination of machine learning algorithms, large language models, robotics, and natural language processing systems designed to mimic human cognitive functions, enabling machines to perform tasks autonomously or with minimal human intervention.

AI transformation refers to the systematic changes in clinical, operational, or organizational processes and business models due to the introduction of AI systems to optimize decision-making, automate tasks, improve patient outcomes, and drive organizational change. This involves identifying opportunities for AI-related innovation, integrating them into processes, and developing strategies to operationalize implementation while ensuring organizational readiness. This is essential for getting health care organizations AI-ready.

Further, in the context of this study, drawing from seminal management and leadership theories, we view leadership as an effective management practice [32]. However, we recognize that leadership roles in health care occur at the clinical, organizational, and systems levels of health systems. At the clinical level, leadership emerges through health care professionals who steer patient care and treatment decisions. At the organizational level, leadership involves middle managers such as unit heads and division leaders guiding health care institutions, administrative units, and personnel toward their goals. At the systems level, leadership encapsulates C-suite leadership responsible for navigating regulatory complexities and organizational and structural silos within complex health systems.

### Eligibility Criteria

The following inclusion and exclusion criteria guided our study: (1) focused on AI in health care, (2) contained an evaluation of leadership, (3) were written in English, (4) were published in a peer-reviewed journal, (5) published between January 2015 and June 2023, and (6) used research.

### Information Sources and Search Strategy

We adopted comprehensive search strategies for the following electronic databases focused on the health care and business literature: MEDLINE (via Ovid), PsycINFO (via Ovid), CINAHL (via EBSCO), Business Source Premier (via EBSCO), and Canadian Business & Current Affairs (via ProQuest). An academic librarian developed these search strategies with input from the research team. We initially conducted the search in Ovid MEDLINE. We then reviewed our search results using the Peer Review of Electronic Search Strategies tool [33], a checklist for comparing, among other things, the types of errors found in articles and the relative fit of articles to the research question before translating the search strategy into other databases using their command language. Our search was limited to articles published from January 2015 (from the first use of AI-powered chatbots in health care [34] to June 2023. We then ran searches in 4 databases and exported the final search results into the EndNote reference management software (Clarivate), and we removed duplicate articles manually. To capture any papers that may have been missed during the search process, we did forward and reverse citation searches of systematic review articles related to AI [35]. However, we did not find any additional articles that met our criteria. Finally, we imported search results to Covidence (Veritas Health Innovation), a review management software for abstract and title screening, full-text screening, and data charting.

### Selection of Sources of Evidence and Data Charting

To minimize selection bias, 2 independent screeners reviewed the titles and abstracts of articles identified via the search against the eligibility criteria using Covidence. We identified articles that met the eligibility criteria for a comprehensive full-text screening. Two independent reviewers then evaluated the full texts against the eligibility criteria using Covidence. In discrepancies between the reviewers, a third reviewer served as the consensus reviewer and used Covidence to resolve conflicts between reviewer 1 and reviewer 2. Following the exclusion of irrelevant articles, we used a predefined data extraction form aligned with our research objectives and guiding questions for systematic data collection. Data extraction categories included data on study characteristics (eg, citations and country); methods (eg, aim, data collection methods, and methodological quality); study context (eg, leadership role, ie, clinical, organizational, or systems); leadership practices (ie, behavior, enablers, and barriers to leadership success); results (ie, main results and author conclusion); and an open-ended reviewer note (ie, capture any relevant information that might aid in the data analysis stage). The data abstraction form was piloted on a random sample of 4 included articles and modified based on feedback from the team. Full data abstraction began only after sufficient agreement had been obtained. Two reviewers independently extracted the data using Covidence, and a third reviewer assessed the data extraction for quality and consensus. Three authors then held a group discussion to resolve any conflicts.

### Risk of Bias Assessment

The focus of scoping reviews is to provide a comprehensive overview of the available literature, identifying the extent, range, and nature of research on a particular topic rather than assessing the methodological quality of individual studies [35]. Therefore, we did not perform risk of bias evaluations on the articles included in compliance with the guidelines for scoping reviews.

### Data Analysis and Synthesis

Our data analysis was guided by a thematic analysis process [36]. To ensure the accuracy of the emerging themes, we conducted our analysis collaboratively in reviewer pairs [35].

We initially analyzed the extracted data using an open-coding method guided by our research questions. Subsequently, we grouped the codes into categories based on the emerging patterns in the data, which we then synthesized into leadership functional domains, capacities, and context.

In the context of our analysis, functional domains refer to distinct areas of responsibility that a leader must effectively manage a task or a role. Capacity, on the other hand, pertains to the abilities—skills, competencies, or behaviors—that a leader must demonstrate to achieve desired goals. Context refers to the environment, conditions, and situational factors that shape and influence leadership practices and decisions.

## Results

### Study Selection

**Figure 1:**
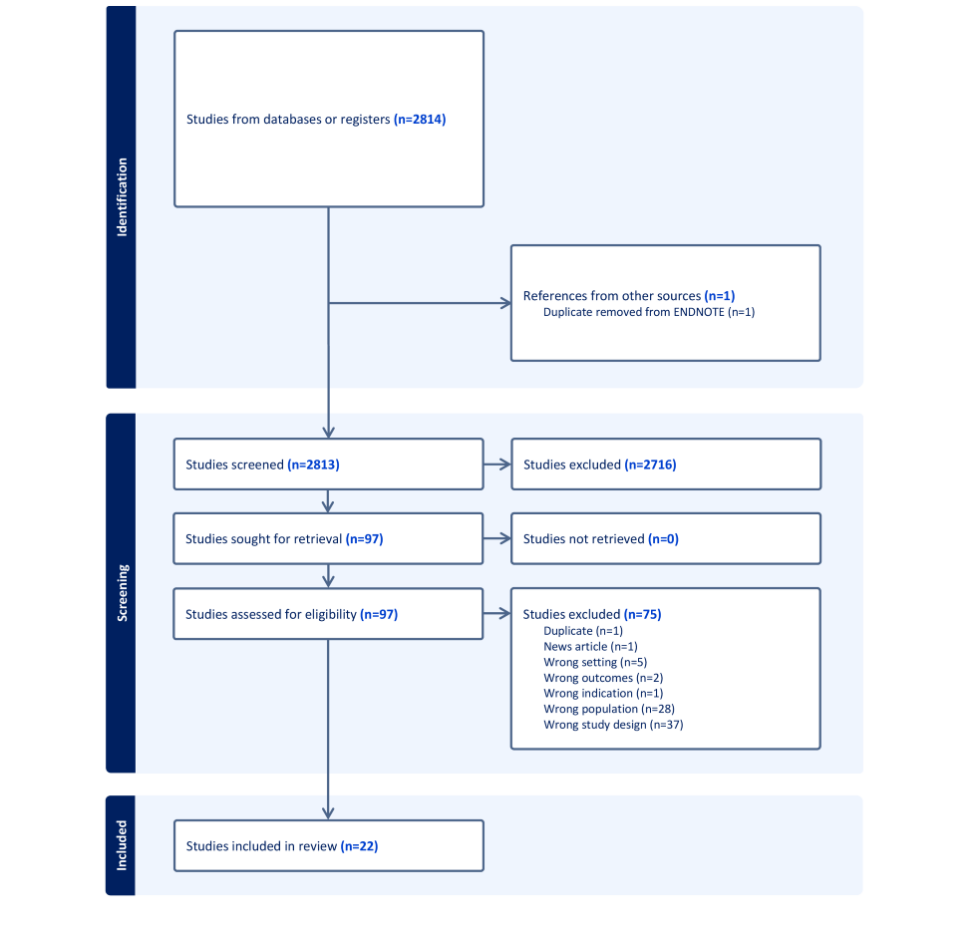
PRISMA (Preferred Reporting Items for Systematic Reviews and Meta-Analyses) flowchart.

As described in Figure 1, the original searches generated 3541 articles published from January 2015 to June 2023. After removing 728 duplicate articles in EndNote, 2813 unique articles were uploaded to Covidence. A total of 2813 relevant studies were then screened using Covidence using the articles’ titles and abstracts. We determined that 97 articles met the criteria for a full-text review for eligibility screening. Within these 97 articles, 75 were excluded as they were opinion articles or commentaries without objective data. After conducting the full-text screening, we found that 22 articles met the final inclusion criteria.

### Study Characteristics

Of the 22 studies identified for final inclusion in our review, 12 involved qualitative methods [37-48] such as interviews and case studies, whereas 4 studies involved mixed methods research [49-52] with a qualitative and quantitative strand. There were 3 narrative reports [53-55] based on document synthesis, and 3 studies involved quantitative methods [56-58] such as surveys. These articles focused on clinical, organizational, and systems leadership and came from Canada, China, Finland, Saudi Arabia, Sweden, the Netherlands, the United Kingdom, and the United States. The included papers addressed a broad array of AI applications in health care, including studies focused on improving workflows, quality of care, patient safety, resource optimization, and patient experience. From a clinical domain, researchers focused on primary care, health care systems, radiology, or global health. From a population perspective, the papers covered leadership from the perspective of primary care physicians, radiologists, nurses, nurse managers, public health professionals, global health professionals, health care entrepreneurs, and health care leaders. Table 1 provides a summary of study characteristics.

**Table 1:** Summary of study characteristics.

Reference	Country	Study context	Leadership level	Theory or framework guiding the research	Study type
Barbour et al [37]	United States	Emergency medicine or medical education	Systems	N/A^a^	Qualitative
Darcel et al [38]	Canada	Primary care	Clinical or systems	Sociotechnological framework	Qualitative
Dicuonzo et al [39]	Canada	Hospital	Organizational or systems	Comprehensive health = technology assessment framework	Qualitative
Dixit et al [53]	Canada	Health care system	Clinical, organizational, or systems	N/A	Narrative report
Ergin et al [58]	Turkey	Nursing	Clinical, organizational, or nursing	N/A	Quantitative
Galsgaard et al [54]	Denmark	Radiology	Clinical	Self-efficacy and professional identity	Narrative report
Ganapathi and Duggal [40]	United Kingdom	Physicians	Clinical	N/A	Qualitative
Gillan [41]	Canada	Radiation medicine and medical imaging technology	Systems or clinical	Normalization Process Theory (NPT)	Qualitative
Hakim et al [49]	Canada	Health care system	Systems or organizational	Health Information and Management Systems Society Adoption Model for Analytics Maturity (AMAM)	Mixed method
Henriksen and Bechmann [42]	Belgium	Technology development	Organizational	Work process and practice-oriented focus	Qualitative
Laukka et al [43]	Finland	Nursing	Organizational, clinical, or nursing	N/A	Qualitative
Li et al [56]	China	Nursing	Organizational, clinical, or nursing	Job Demand-Control-Support (JDCS) model	Quantitative
Morley et al [50]	United Kingdom	Global health	Systems or global health	N/A	Mixed method
Nasseef et al [57]	Saudi Arabia	Health care organization	Systems or public health	Cognitive Fit Theory (CFT)	Quantitative
Olaye and Seixas [44]	United States	Health care startups	Systems or digital health startup	N/A	Qualitative
Petersson et al [45]	Sweden	Health care system	Organizational or systems	N/A	Qualitative
Ronquillo et al [46]	International	Nursing	Systems, clinical, or nursing	N/A	Qualitative
Sawers et al [55]	International	Sustainable development goals—eye health	Systems or global health	N/A	Narrative review
Strohm et al [47]	Netherland	Radiology	Clinical	Nonadoption, Abandonment, Scale-up, Spread, and Sustainability (NASSS) Framework for new medical technologies in health care organizations.	Qualitative
Upshaw et al [48]	Canada	Primary care	Systems	Sittig and Singh’s model for studying Health Information Technology (HIT) in complex adaptive health systems	Qualitative
Willis et al [51]	United Kingdom	Primary care	Clinical	O*NET classification of occupational tasks	Mixed method
Yang et al [52]	China	Hospital	Organizational or systems	Technology-Organization-Environment (TOE) Framework	Mixed method

^a^N/A: not applicable.

### Leadership Tasks Essential for AI Transformation in Health Care

We mapped the themes from the included studies across 4 functional domains of leadership task responsibility—technological (AI innovation), strategic (vision and alignment), operational (process and oversight), and organizational (culture and work environment).

The technological functional domain garnered the most significant attention in the literature. The core themes that emerged under the technological domain primarily focused on applying subject matter expertise and AI technical skills to effectively identify AI opportunities, as well as to foster an innovation mindset to develop, tailor, and seamlessly implement AI-driven solutions to address key AI opportunities within health care organizations.

Within the strategic functional domain, the literature underscored the importance of change management and communication as strategic tools for consensus and collaboration related to the AI transformation process. Another core theme that emerged focused on the critical importance of integrating AI solutions into the existing clinical care processes. This strategic alignment is essential for getting support from the staff and ensuring smooth operations of patient care outcomes while embracing the potential of AI solutions. Although the significance of talent strategy related to the recruitment and retention of AI technical expertise within organizations was mentioned, it was not widely seen across the included papers.

Table 2 provides a summary of how the technological and strategic functional domains map across the papers and provides key themes that emerged with the domain area.

**Table 2:** Leadership competencies related to technical and strategic functional domains.

Reference	Functional domain	Key themes				Functional domain		Key themes		
	Technological	Subject matter expertise	Technical skills	Innovation mindset	Strategic		Change		Communication	Alignment
Barbour et al [37]	✓	✓	✓							
Darcel et al [38]	✓		✓		✓				✓	
Dicuonzo et al [39]	✓		✓		✓				✓	
Dixit et al [53]					✓		✓		✓	
Ergin et al [58]	✓	✓	✓							
Galsgaard et al [54]	✓	✓			✓		✓		✓	
Ganapathi and Duggal [40]	✓	✓	✓		✓					✓
Gillan [41]	✓	✓			✓				✓	
Hakim et al [49]	✓	✓	✓		✓				✓	✓
Henriksen and Bechmann [42]	✓	✓	✓		✓				✓	
Laukka et al [43]	✓	✓								
Li et al [56]	✓			✓	✓		✓			
Morley et al [50]	✓	✓	✓							
Nasseef et al [57]	✓				✓					✓
Olaye and Seixas [44]	✓	✓			✓					✓
Petersson et al [45]	✓		✓		✓				✓	✓
Ronquillo et al [46]	✓	✓	✓		✓		✓			
Sawers et al [55]	✓			✓	✓				✓	
Strohm et al [47]	✓			✓	✓		✓		✓	✓
Upshaw et al [48]	✓	✓	✓							
Willis et al [51]	✓			✓	✓		✓			✓
Yang et al [52]	✓			✓	✓		✓			✓

Emerging evidence in the operational functional domain highlights leaders’ need to navigate ethical and risk management issues by establishing robust governance structures prioritizing patient data privacy and security while ethically integrating AI technologies within existing workflows. Additionally, the literature emphasizes that implementing AI in health care will require leaders to ensure new AI solutions comply with existing regulatory and control systems. The literature highlighted that leaders need to pay attention to process agility through continuous monitoring to ensure AI solutions can adapt to contextual changes.

Finally, the organizational functional domain emerges from the thematic analysis as a pivotal area for AI leadership. The literature emphasizes the importance of stakeholder engagement in building collaboration. Furthermore, it underscores the importance of decision makers’ sense-making to enhance their trust in AI opportunities and ensure that AI integration is supported by individuals across the organization. Further, the literature underscored the importance of organizational culture readiness to support physicians and nurses through protected time and incentive pay to engage, innovate, and adopt AI solutions. Table 3 provides a summary of how operational and organizational functional domains map across the papers.

**Table 3:** Leadership competencies related to operational and organizational functional domains.

Author	Functional domain	Key themes				Functional domain		Key themes		
	Operational	Ethical and risk management	Regulatory compliance	Process agility	Organizational		Stakeholder engagement or collaboration		Trust and sense-making	Organizational culture and readiness
Barbour et al [37]										
Darcel et al [38]	✓	✓	✓		✓		✓			✓
Dicuonzo et al [39]	✓				✓		✓			
Dixit et al [53]	✓	✓					✓			
Ergin et al [58]	✓	✓								
Galsgaard et al [54]					✓		✓			
Ganapathi and Duggal [40]					✓		✓			✓
Gillan [41]	✓	✓		✓	✓		✓		✓	
Hakim et al [49]	✓	✓	✓	✓	✓		✓			
Henriksen and Bechmann [42]					✓		✓		✓	
Laukka et al [43]	✓	✓			✓					
Li et al [56]	✓			✓						
Morley et al [50]	✓	✓	✓		✓		✓			
Nasseef et al [57]	✓	✓					✓			
Olaye and Seixas [44]	✓	✓	✓		✓		✓			
Petersson et al [45]	✓	✓	✓		✓		✓			✓
Ronquillo et al [46]	✓	✓			✓		✓			
Sawers et al [55]	✓	✓	✓		✓		✓			✓
Strohm et al [47]	✓				✓		✓			✓
Upshaw et al [48]	✓	✓								
Willis et al [51]	✓				✓					
Yang et al [52]	✓		✓		✓		✓		✓	

### Leadership Skills and Behaviors for Preparing Health Care Organizations for AI Transformation

We categorized the themes related to skills and behaviors into 3 essential capacities that a leader must demonstrate to achieve desired goals—technical capacity, adaptive capacity, and interpersonal capacity. Technical capacity encompasses (1) AI literacy, (2) subject matter knowledge, (3) change leadership skills, and (4) innovation mindset to identify AI innovation opportunities. The interpersonal capacity involves several vital facets such as (1) the ability to foster partnerships among diverse stakeholders, (2) the ability to comprehend diverse stakeholder perspectives and deftly influence adoption, (3) the ability to build trust and collaboration, (4) self-awareness and humility to assemble teams with complementary skills, and (5) the integrity and accountability to embody ethical principles. The adaptive capacity encompasses (1) the foresight and sense-making abilities to discern emerging technologies and their implications within the health care sphere; (2) the agility to identify and capitalize on transformative opportunities, swiftly adapting and aligning strategies with evolving contexts; and (3) systems thinking to enable an understanding of how elements interconnect and how changes in 1 area can reverberate throughout the entire system.

### Contextual Factors Influencing Leadership in AI Transformation

The emerging themes from our review reveal that dynamic environmental and situational factors, including regulatory, technology, and organizational contexts, shape AI transformation within health care organizations. For instance, the regulatory context and frameworks related to health professions and health care organizations play a critical role in how AI can be integrated within the organizations. Similarly, the technology context such as the availability of AI technical talent, the retention of technical expertise, the dynamic nature of AI maturity, and the presence of incentives and technological resources for AI innovation or adoption will significantly influence a leader’s ability to effectively drive AI readiness. Finally, the organization context is a critical influence on leaders’ capacity for AI adoption and implementation. Organizations that promote and reward innovation and that have transparent communication practices shape leaders’ ability to pursue AI opportunities.

### Strategies for Empowering Health Care Leaders to Facilitate AI Transformation

For the technological domain, the included papers discussed approaches such as upskilling clinical experts with the necessary AI technical skills and ensuring the presence of specialized experts, such as computer scientists, to enable the subject matter experts to develop, test, and seamlessly integrate AI solutions. Further, the papers discussed collaborative strategies such as clinicians and computer scientists working together to effectively identify AI opportunities and develop, adopt, and implement AI solutions in clinical or operational areas.

For the strategic domain, organizational support was essential in supporting leaders to assess and identify AI opportunities that strategically align with organizational priorities and develop strategies to ensure AI transformation garners support from key stakeholders within the complex regulatory and environmental contexts. The literature also highlighted the competition for AI talent in health care and emphasized the significance of talent retention strategies to preserve the organization’s AI technical expertise.

Then, in the operational domain, the emphasis was on establishing governance structures to continuously monitor data quality, patient privacy, and patient care experiences and assess the feasibility and financial implications of AI transformation. These governance structures ensure effective oversight and management of AI initiatives within health care organizations.

Finally, for the organizational domain, the focus was on the pivotal role of organizational culture in AI leadership. Leaders require organizational support to cultivate an environment that fosters innovation and actively incentivizes clinical leaders, such as physicians and nurses, through protected time and incentive pay to innovate and adopt AI solutions. Transparent decision-making processes related to AI solutions are essential cultural elements that build trust in AI systems and promote collaboration among the diverse stakeholders involved in AI transformation within health care organizations.

## Discussion

### Principal Findings

**Figure 2:**
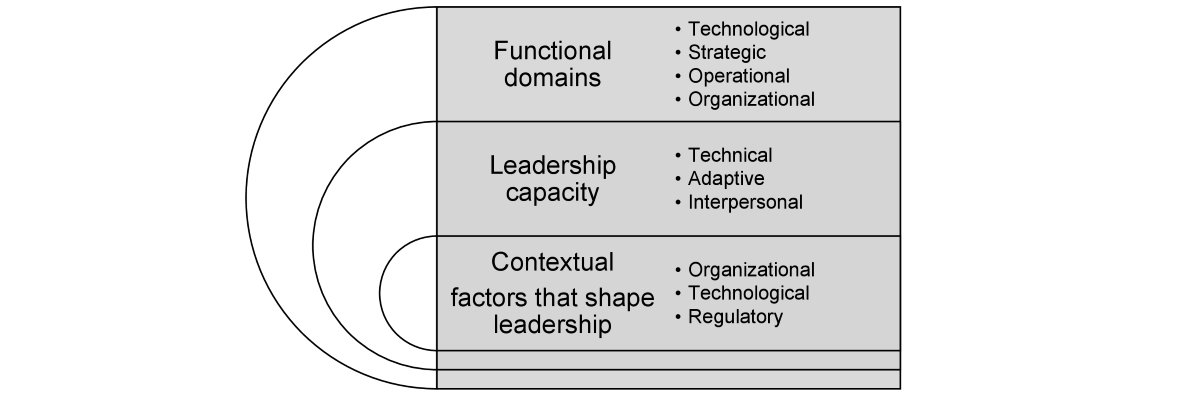
Multidimensional leadership framework for artificial intelligence (AI) transformation.

The purpose of a scoping review is not to draw definitive conclusions but to map the literature, identify emerging patterns, and develop critical propositions. As described in Figure 2, analysis of current literature shows that leading organizations toward AI transformation requires multidimensional leadership. As such, health care organizations need to engage leaders in the technological, strategic, operational, and organizational domains to facilitate AI transformation in their organizations. Further, the reviewed papers suggest that individuals in AI-related leadership roles need to demonstrate (1) technical capacity to understand the technology and innovation opportunities, (2) adaptive capacity to respond to contextual changes, and (3) interpersonal capacity to navigate the human aspects of the AI transformation process effectively. Furthermore, our study illuminates that leaders in the AI-related leadership roles need to navigate regulatory context, the dynamic nature of changing technology context, and organization context.

### Prior Research

Health care organizations are marked by multifaceted interdependencies among medical facilities, health care providers, patients, administrative units, technology, and the regulatory environment. Therefore, the leadership required for AI transformation—which includes identifying AI opportunities, implementing AI solutions, and achieving full-scale AI adaptation—is not a static role but a continuous and dynamic process. Effective leadership involves the capacity to continuously identify opportunities for AI transformation, influence the thoughts and actions of others, and navigate the complex dynamics of the health care setting and AI technology landscape simultaneously. However, the current literature has not fully articulated this multidimensionality, often focusing on leadership through a linear approach.

Further, multiple situational factors can shape AI transformation. First, the rapid growth of AI technologies introduces an element of uncertainty, making it challenging to anticipate the long-term impact and sustainability of specific AI solutions [6]. Second, AI implementation involves many stakeholders, from technical experts and domain specialists to clinicians, administrators, patients, vendors, and regulatory bodies. Each stakeholder group brings its unique perspectives, priorities, and control systems into the equation, necessitating leaders to navigate competing values, trade-offs, and paradoxes [27]. Third, once alignment is achieved, the integration of AI within an organization triggers a need for a cultural shift, altering work practices and decision-making processes [38,59]. Fourth, the effectiveness of AI solutions hinges on the availability of high-quality data for informed insights and decision-making. When implementing solutions originally developed within different contexts, local organizations must ensure data integrity and the solution’s adaptability to the organization’s unique context [18]. This challenge is compounded by emerging regulatory frameworks, which add a layer of complexity. Ensuring compliance and the responsible use of AI technologies has become a critical consideration [29,50,60]. Finally, introducing AI may provoke resistance from employees concerned about job displacement or disruptions to established workflows. This problem is further compounded when an organization transitions toward integrating multiple AI systems, as these changes can lead to periods of chaos and confusion [59].

Emerging key opinions and evidence from outside the health domain indicate that leaders must possess an understanding of data quality nuances, assess process risks, and manage AI as a new team member. Additionally, leaders should have a firm grasp of technology, articulate clear business objectives, define precise goals, uphold a long-term vision, prepare their teams for AI transformation, manage data resources effectively, and foster organizational collaboration [3,61-67].

Our findings on the leadership required for AI transformation in health care organizations reinforce this multidimensionality of leadership to effectively navigate the complexities of AI transformation and successfully leverage its potential to drive transformative change. Leaders must operate across different functional domains—technological, strategic, operational, and organizational—while demonstrating technical, adaptive, and interpersonal capacities.

Further, our findings show contingency leadership theories, complexity theory, and transformational leadership theory as relevant theoretical domains for further explaining the different facets of leadership behaviors needed to navigate the multidimensionality of leadership required for AI transformation.

Contingency theories suggest that leadership effectiveness depends on situational factors, which should be considered in future AI implementation studies in the context of AI adaptation and integration within health care organizations [68,69]. Complexity theory provides a framework for examining leadership behaviors in interconnected, dynamic environments where leaders must balance innovation and stability and demonstrate an adaptive approach to challenges, characterized by uncertainty and change [70-73]. Transformational leadership theory emphasizes motivating, empowering, and developing others by fostering trust and collaboration while challenging the status quo to drive organizational change and innovation [74,75]. These theories should be considered in future AI implementation studies within health care organizations.

Future research and training programs related to AI in health care should examine the leadership required for AI transformation through the lens of multidimensionality, providing insights into the interrelatedness of functional domains, leadership capacities, and contextual enablers and barriers, while exploring the key theoretical domains related to contingency, complexity, and transformational leadership to further understand the interpersonal dynamics shaping AI transformation in health care.

### Limitations

Some limitations to our scoping review are worth noting. First, given the contextual variability in the included studies and the methodological variations, we could not establish firm correlations about specific leadership domains, capacities, and contextual factors; the effectiveness of leadership approaches; or the moderating effects of contextual factors. Consequently, we have presented only the overarching emergent themes.

Second, our study is limited by the significant variation in conceptual definitions of leadership and leadership competencies found in the current literature, which often lacks more standardized definitions or instruments for measurement. This variation caused conceptual inconsistencies. We addressed the inconsistencies by clearly defining what constitutes a functional domain, capacity, and context before our data analysis to address this. We iteratively coded the data into themes to ensure all relevant aspects were captured.

Third, our search strategy focused on MEDLINE-indexed journals, which may exclude some newer journals indexed in PubMed but not yet in MEDLINE. While this might limit the capture of the very latest advancements in digital health, it does not diminish the robustness of the review. Fourth, we retrieved only articles written in English, which possibly limited the comprehensiveness of our findings. Fifth, we looked at AI as a system and did not look at the relationship between the implementation of different types of AI tools and leadership behaviors which was beyond the scope of our review. Finally, our analysis used an inductive approach and was not informed by a predetermined theory to aid the mapping of the literature. This may have limited our analysis in capturing different elements of an umbrella theory.

### Recommendations for Future Design and Research

Leading organizations toward AI transformation is an adaptive challenge influenced by a myriad of interwoven situational factors that create a dynamic and intricate environment. The body of literature related to AI in health care is rapidly expanding, and the recommendations imparted by this review, alongside the multidimensional leadership framework (Figure 2), stand poised to guide research and practice to empower health care organizations in their AI transformation journey. Future research on AI transformation, which includes innovation identification, implementation, and scaling, can use this framework to understand the role of leadership in driving successful outcomes.

Further, future research must undergo methodological expansion by embracing qualitative and mixed methods approaches to illuminate the intricate temporal aspects of AI transformation and corresponding evolving leadership behaviors. 

### Conclusions

In summary, emerging evidence shows that multidimensional leadership plays crucial role in AI transformation in health care organization. Leaders must adeptly balance technology opportunities while demonstrating unwavering empathy for stakeholder needs and nimble adaptability to accommodating the ever-changing contextual landscape, which encompasses the regulatory frameworks, the evolution of technology, and the organization’s priorities.

## Appendix

Multimedia Appendix 1 Updated PRISMA (Preferred Reporting Items for Systematic Reviews and Meta-Analysis) checklist.

## Abbreviations

AI: artificial intelligence
PRISMA-ScR: Preferred Reporting Items for Systematic Reviews and Meta-Analysis extension for Scoping Reviews

## Data Availability

The main study data are the data extraction materials and quality ratings of included papers, most of which are included in the study tables. The data sets generated and analyzed during this study are available from the corresponding author on reasonable request.
